# Patterns of metastatic spread and tumor burden in unselected cancer patients using PET imaging: Implications for the oligometastatic spectrum theory

**DOI:** 10.1016/j.ctro.2024.100724

**Published:** 2024-01-09

**Authors:** Sebastian M. Christ, Kaspar Pohl, Jonas Willmann, Philip Heesen, Astrid Heusel, Maiwand Ahmadsei, Anja Kühnis, Eugenia Vlaskou Badra, Urs J. Muehlematter, Michael Mayinger, Panagiotis Balermpas, Nicolaus Andratschke, Nicholas Zaorsky, Martin Huellner, Matthias Guckenberger

**Affiliations:** a Department of Radiation Oncology, University Hospital Zurich, University of Zurich, Zurich, Switzerland; b Faculty of Medicine, University of Zurich, Zurich, Switzerland; c Department of Nuclear Medicine, University Hospital Zurich, University of Zurich, Zurich, Switzerland; d Center for Proton Therapy, Paul Scherrer Institute, ETH Domain, Villigen, Switzerland; e Department of Radiation Oncology, University Hospitals Seidman Cancer Center, Case Western Reserve University School of Medicine, Cleveland, OH, USA

**Keywords:** Polymetastasis, Tumor burden, PET imaging

## Abstract

**Introduction and background:**

Metastatic disease has been proposed as a continuum, with no clear cut-off between oligometastatic and polymetastatic disease. This study aims to quantify tumor burden and patterns of spread in unselected metastatic cancer patients referred for PET-based staging, response assessment of restaging.

**Materials and methods:**

All oncological fluorodeoxyglucose (FDG-) and prostate-specific membrane antigen (PSMA-) positron emission tomography (PET) scans conducted at a single academic center in 2020 were analyzed. Imaging reports of all patients with metastatic disease were reviewed and assessed.

**Results:**

For this study, 7,000 PET scans were screened. One third of PET scans (n = 1,754; 33 %) from 1,155 unique patients showed presence of metastatic disease from solid malignancies, of which 601 (52 %) and 554 (48 %) were classified as oligometastatic (maximum 5 metastases) and polymetastatic (>5 metastases), respectively. Lung and pleural cancer, skin cancer, and breast cancer were the most common primary tumor histologies with 132 (23.8 %), 88 (15.9 %), and 72 (13.0 %) cases, respectively. Analysis of the number of distant metastases showed a strong bimodal distribution of the metastatic burden with 26 % of patients having one solitary metastasis and 43 % of patients harboring >10 metastases. Yet, despite 43 % of polymetastatic patients having >10 distant metastases, their pattern of distribution was restricted to one or two organs in about two thirds of patients, and there was no association between the number of distant metastases and the number of involved organs.

**Conclusion:**

The majority of metastatic cancer patients are characterized by either a solitary metastasis or a high tumor burden with >10 metastases, the latter was often associated with affecting a limited number of organs. These findings support both the spectrum theory of metastasis and the seed and soil hypothesis and can support in designing the next generation of clinical trials in the field of oligometastatic disease.

## Highlights

• Metastatic disease is hypothesized to represent a disease continuum.
• This study quantified tumor burden and patterns of spread using PET/CT imaging.
• Patients were found to have either few (<5) or many (>10) distant metastases.
• Our findings support both the spectrum theory and seed and soil hypothesis.

## Introduction and background

Metastatic disease characterizes a situation of systemic cancer spread throughout the body. Many cancer patients will ultimately suffer from metastatic disease and metastasis is the cause of more than 90 % of cancer mortality [1]. Metastatic disease is heterogeneous, both on a genetic and a clinical level: Different genetic drivers of the metastatic process have been identified in various primary cancers, and the significance of the tumor microenvironment including the immune system has been widely recognized [2]. A better understanding of cancer biology allows clinicians to increasingly individualize treatment options, yet only one metastatic sub-staging tumor category – stage IV cancer – continues to exist in the TNM staging systems of many cancer types. This has been argued to be an insufficient and unsatisfactory situation to describe this heterogeneous group of patients [3].

One differentiation, is the distinction between oligo- and polymetastatic disease states [4]. Definitions of the two metastatic states vary, yet a maximum of three or five imaging-defined distant metastases are commonly used as cut-off criteria to distinguish the two [5], [6], [7]. In the absence of clinically viable biomarkers, other factors such as volumetric tumor burden [8], metastatic velocity [9] and/or clinical phenotypes have been proposed as prognostic factors [3], [10]. The benefit of definitive local therapy has been suggested in several small phase II trials to date [11], [12], [13], [14], [15]. However, if the paradigm of the spectrum theory, according to which cancer is a spectrum of metastatic diseases without a clear cut-off between oligo- and poly-metastatic disease, turns out to be true, then patients with more than five distant metastases might also achieve a (potentially smaller) benefit from definitive local therapy to all macroscopic cancerous lesions [4], [16]. This concept is currently being evaluated in the SABR COMET 10 trial (NCT03721341), where the impact of addition comprehensive definitive local therapy to 4–10 macroscopic cancer lesions to standard-of-care is being investigated [17]. Moreover, the ongoing ARREST trial (NCT04530513) assesses definitive local therapy in polymetastatic patients with more than 10 distant metastases in a phase I, modified 3 + 3 design [18].

However, little is known about the detailed pattern of spread in unselected metastatic cancer patients, which were identified through imaging screening and not treatment logs. In light of the limited literature elucidating the epidemiology of widespread distant metastasis [3], [19], [20], this is the first study to perform a detailed analysis of metastatic patterns using positron emission tomography (PET) imaging, which nowadays can be regarded as gold standard for the detection of oligometastatic states, even though many historical trials investigating oligometastasis did previously not employ PET imaging. The purpose of this research study is to contribute to this expanding research field by assessing metastatic patterns of solid organ tumors, quantifying tumor burden in metastatic cancer patients, and discussing our results in the context of the spectrum theory and seed and soil hypothesis of metastatic disease.

## Materials and methods

### Patient population

For this single-center retrospective observational study, all oncological fluorodeoxyglucose (FDG-) and prostate-specific membrane antigen (PSMA-) PET scans conducted at our comprehensive cancer center between January and December 2020 were screened. Indications for ordering a PET scan at our institution included initial cancer staging, treatment response monitoring, re-staging, and/or detection of tumor recurrence. PET scans of patients were included into the analysis if they had a malignant extra-cranial solid cancer diagnosis, and if any metastases were visible on PET imaging. In the case of multiple PET scans per patient, the first PET scan as included into the analysis. PET scans from patients with hematologic malignancies or primary brain tumors were excluded from the analysis.

### *Data* collection *process*

Variables of interest included age at date of PET scan, gender, primary diagnosis code, type of PET scan, date of scan, presence of metastasis, number of metastases, and the location of metastases. PET imaging reports were pre-screened for the wording of “metastatic disease” employing previously tested natural language processing (NLP) algorithms, i.e., a regex-based rule system. Subsequently, the pre-screened data was manually reviewed and complemented from the imaging report and/or the electronic medical records by one researcher (KP), and selected examples were cross-checked by another researcher (SMC, PH or MG). Where imaging reports were inconclusive, the PET scans were individually reviewed (AH and SMC). Equivocal lesions, which nuclear medicine physicians marked as indeterminate (inflammatory vs. infectious vs. cancerous), were only included into the metastasis count, if the diagnosis was ascertained by tissue analysis, for example, through a biopsy.

### Categorization of metastases

A cut-off value of maximum five distant metastases was used to distinguish oligo- and polymetastatic disease states [5]. Whenever the imaging report did not explicitly state the number of distant metastases, an attempt was made to manually count the number of metastases based on the imaging report. Patient cases where imaging reports included expressions such as “disseminated tumor spread in multiple organs” or “wide-spread extra-cranial metastasis”, for instance, or where the nuclear medicine physician made no attempt of quantifying diffusely present metastasis, we reviewed the PET scan images counted every individual metastatic lesion. Evidence for any type of carcinomatosis in a polymetastatic patient was regarded as uncountable and consequently added under the “>10 distant metastases” category. For a subset of oligometastatic patients, concurrent brain scans were available for review.

### Data and statistical analysis

All data was collected and recorded in Microsoft® Excel® (Version 16.0). Descriptive summary statistics and Chi-squared test statistics were calculated using the statistical software package STATA® (v.16.0). Graphs were created using Microsoft® Powerpoint® (Version 16.0). Our study was approved by the Swiss Cantonal Ethics Committee (BASEC ID# 2018–01794) and the hospital-internal Data Governance Board (DUP-42).

## Results

### Imaging scans

In 5,773 (82 %) of 7,000 oncological PET scans, the imaging report contained a cancer diagnosis. The large majority (n = 5,358; 93 %) of those scans originated from patients with a solid extra-cranial malignancy. Of those, 1,754 (33 %) scans from 1,155 unique patients showed presence of metastatic disease, whereof 518 (45 %) harbored more than five extra-cranial distant metastases and were therefore classified as exhibiting a polymetastatic disease state. Out of 637 (55 %) patients with extra-cranial oligometastatic disease, 36 (6 %) were rendered polymetastatic after brain scan review, resulting in a total of 554 (48 %) truly polymetastasized patients.

### Patient demographics and primary tumor histologies

The median age of polymetastasized patients at the time of PET scan was 66 years (interquartile range (IQR), 57–74). A proportion of 43.1 % (n = 239) polymetastatic patients were female. Lung and pleural cancer, skin cancer, and breast cancer were the most common primary tumor histologies with 132 (23.8 %), 88 (15.9 %), and 72 (13.0 %) cases, respectively. Pancreas, liver and gallbladder, cancer of unknown primary (CUP) and upper gastrointestinal tract (GIT) cancers were the least common with 26 (4.7 %), 23 (4.2 %) and 12 (2.2 %) cases, respectively (Table 1).

**Table 1:** Metastatic disease spectrum by age, gender and primary cancer.

**Parameters**	**1**–**5 distant metastases**	**6**–**10 distant metastases**	**>10 distant metastases**	**P-value**
Age at scan, median (IQR)	65 (56–73)	68 (56–75)	66 (57–74)	0.731
Female gender, n (%)	213 (35)	18 (32)	221 (45)	0.062
Frequency of metastatic state, n (%)				0.303
Lung & pleura^1^	160 (27)	12 (21)	120 (24)	
Skin^2^	116 (19)	9 (16)	79 (16)	
Breast	47 (8)	6 (11)	66 (13)	
Prostate	80 (13)	9 (16)	52 (11)	
Genitourinary^3^	27 (4)	5 (9)	37 (7)	
Head & Neck	53 (9)	5 (9)	35 (7)	
Colorectal	39 (6)	4 (7)	26 (5)	
Pancreas, liver, and gallbladder	37 (6)	5 (9)	21 (4)	
Cancer of unknown primary	8 (1)	0 (0)	23 (5)	
Upper GIT^4^	13 (2)	1 (2)	12 (2)	
Other^5^	21 (3)	0 (0)	23 (5)	
Total	601 (100)	60 (100)	494 (100)	

*Abbreviations:* GIT = Gastrointestinal tract; IQR = Interquartile range; PET = Positron emission tomography.

1 Includes small-cell lung cancer (SCLC), non-small cell lung cancer (NSCLC) and mesothelioma.

2 Includes melanoma and squamous cell carcinoma;

3 Excluding prostate cancer;

4 Excluding pancreatic cancer;

5 Includes thyroid cancers and sarcomas.

### Distant metastasis distribution

**Fig. 1:**
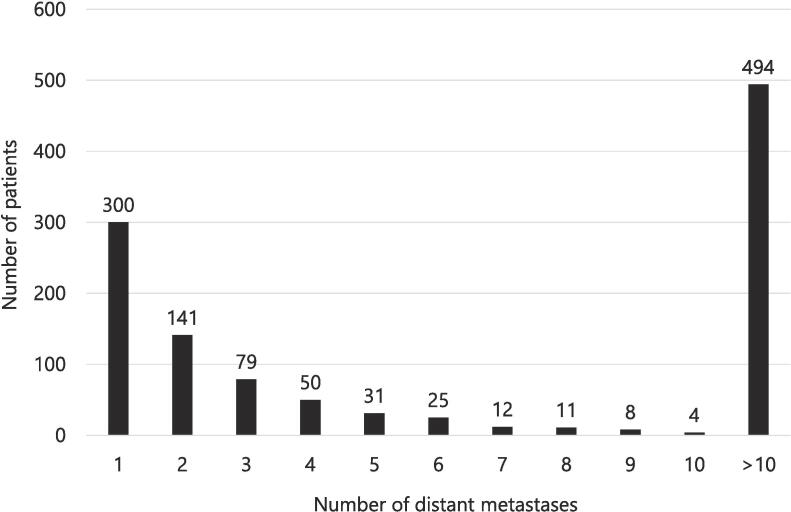
Distribution of number of distant metastases of all metastatic patients (n = 1,155). *Note:* Absolute number of distant metastases from n = 601 oligo- and n = 554 polymetastasized patients based on extra-cranial PET imaging and concurrent brain scans for oligometastatic patients where available.

Across all 1,155 metastatic patients, most patients had a solitary (n = 300/n = 1,155; 26 %) or >10 distant metastases (n = 494/n = 1,155; 43 %), with the remainder of patients having between two and ten lesions on PET imaging (n = 361/n = 1,155; 31 %) (Figure 1). The same distribution pattern manifested when evaluating all primary tumor histologies separately.

### Polymetastatic patterns and total tumor burden

Of 554 patients with polymetastatic disease, 494 (89 %) had >10 distant metastases and only 60 (11 %) had between 6 and 10 distant metastases (Table 1). In almost two thirds of patients, all distant metastases were distributed over maximum two organs: the clinical situations with all metastases located in one organ or two organs were observed in n = 182 (33 %) and n = 155 28 % patient, respectively (Table 2).

**Table 2:** Number of metastatic sites and extent of organ involvement.

**Parameters**	**Data**
Number of distant metastases in polymetastatic patients, n (%)	n = 554
6 metastases	25 (5)
7 metastases	12 (2)
8 metastases	11 (2)
9 metastases	8 (1)
10 metastases	4 (1)
>10 metastases	509 (92)
Number of involved metastatic sites, n (% of total polymetastatic patients)	n = 554
1 organ	182 (33)
2 organs	155 (28)
3 organs	114 (21)
4 organs	51 (9)
5 organs	18 (3)
>5 organs	34 (6)

### Statistical associations characterizing patterns of polymetastasis

**Fig. 2:**
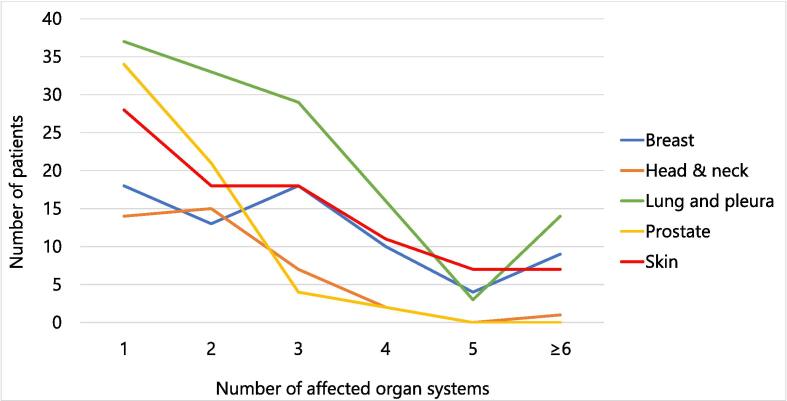
Number of affected organ systems stratified by five most prevalent primary tumor histologies in this cohort. *Note:* “Lung” includes small-cell and non-small cell lung cancer; “Skin” includes melanoma and squamous cell carcinoma.

Number of distant metastases was neither significantly associated with number of organ systems affected by distant metastasis (p = 0.299) nor with primary tumor histology (p = 0.492). Primary tumor histology, however, was significantly associated with number of organ systems affected by distant metastasis (p < 0.001). Polymetastatic disease with distant metastasis present in a single organ system was most common in prostate cancer patients with 56 % (n = 34/n = 61) and patients with thyroid cancer and sarcomas with 52 % (n = 12/n = 23). Scenarios of five or six organs harboring distant metastases were most common in patients with skin cancer (n = 14/88; 16 %), breast cancer (n = 9/n = 72; 13 %), and lung and pleura cancer (n = 17/132; 13 %) (Table 3; Figure 2).

**Table 3:** Number of affected organ systems stratified by primary tumor histology.

**Primary // Number of organs**	**1**	**2**	**3**	**4**	**5**	**≥6**	**Total**	**p-value**
Breast, n (%)	18 (25)	13 (18)	18 (25)	10 (14)	4 (5)	9 (13)	72 (100)	<0.001
Cancer of unknown primary, n (%)	7 (30)	6 (26)	6 (26)	2 (1)	2 (1)	0 (0)	23 (100)	
Colorectal, n (%)	8 (25)	14 (44)	4 (13)	2 (6)	1 (3)	1 (9)	30 (100)	
Genitourinary, n (%)^1^	12 (26)	14 (46)	9 (21)	4 (9)	2 (5)	1 (2)	42 (100)	
Head & neck, n (%)	14 (35)	15 (38)	7 (18)	2 (5)	0 (0)	1 (3)	40 (100)	
Lung and pleura, n (%)^2^	37 (28)	33 (25)	29 (22)	16 (12)	3 (2)	14 (11)	132 (100)	
Pancreas, liver, gallbladder, n (%)	7 (27)	9 (35)	7 (27)	2 (8)	0 (0)	1 (4)	26 (100)	
Prostate, n (%)	34 (56)	21 (34)	4 (7)	2 (3)	0 (0)	0 (0)	61 (100)	
Skin, n (%)^3^	28 (32)	18 (21)	18 (21)	11 (12)	7 (8)	7 (8)	88 (100)	
Upper GIT, n (%)^4^	1 (8)	2 (15)	7 (54)	2 (15)	0 (0)	1 (8)	13 (100)	
Other, n (%)^5^	12 (52)	5 (22)	2 (9)	2 (9)	1 (4)	1 (4)	23 (100)	
Total, n (%)	178 (32)	150 (27)	111 (20)	54 (10)	20 (4)	37 (7)	554 (100)	

*Abbreviations:* GIT = Gastrointestinal tract.

1 Excluding prostate cancer;

2 Includes small-cell lung cancer (SCLC), non-small cell lung cancer (NSCLC) and mesothelioma;

3 Includes melanoma and squamous cell carcinoma;

4 Excluding pancreatic cancer;

5 Includes thyroid cancers and sarcomas.

## Discussion

To the best of our knowledge, this is one of the first studies to perform a detailed analysis of the patterns of metastatic spread and tumor burden in a large cohort of unselected cancer patients with polymetastatic disease. We assume a low patient selection bias, as our analysis is based on consecutive diagnostic PET imaging scans and not based on an indication for any therapeutic intervention. In 1,155 unique cancer patients with distant metastases, a statistically unexpected disease pattern was observed. Using a binary definition of oligo- vs. polymetastatic disease with a cut-off of n = 5 metastases to differentiate between the two states, 52 % and 48 % of patients were classified as having oligo- and polymetastatic disease, respectively. Analysis of the number of distant metastases showed a strong bimodal distribution of the metastatic burden with 26 % of patients having one solitary metastasis and 43 % of patients harboring >10 metastases. Yet, despite 43 % of polymetastatic patients having >10 distant metastases, their pattern of distribution was restricted to one or two organs in about two thirds of patients, and there was no association between the number of distant metastases and the number of involved organs.

The oligometastatic paradigm was first proposed by Hellman and Weichselbaum in 1995. This concept describes the existence of an intermediate state between locoregional and widespread cancer [16]. The oligometastatic disease state is based on the spectrum theory of cancer metastasis, according to which different metastatic states are characterized by their unique biology, including genetic, epigenetic, and immunogenic determinants, and consequently manifest clinically with varying number of metastatic lesions, distinct tumor burden and symptoms [21]. As this theory will be scrutinized in the future both on a biological and clinical level, data on the prevalence and patterns of what is currently referred to as polymetastatic disease will be relevant to inform clinical investigations [22]. To this day, detailed tumor burden reporting in clinical trials remains suboptimal [23], [24].

Our findings are particularly relevant for the design of future oligometastasis clinical trials, as our data shows that increasing the threshold for the maximum number of distant metastases as an inclusion criterion for trials will have only little impact on patient recruitment. The SABR COMET 10 trial (NCT03721341), designed for patients with 4–10 distant metastases, effectively focuses on a small subgroup of metastatic patients, as this number of metastases were rarely detected on PET imaging in this consecutive patient cohort. Moreover, this finding was independent from the primary tumor histology, which might support the value of conceptualizing and conducting proof-of-principle oligometastatic disease trials in a disease-agnostic fashion, although oncological results might vary depending on histology.

Even though the largest subgroup of polymetastatic disease patients was the one with >10 distant metastases, in about two thirds of these patients all metastatic lesions were located in only one or two organ systems. This is in agreement with Paget’s ‘seed and soil’ hypothesis, according to which metastasis is the result of the complex interplay and ultimately the compatibility of the metastatic tumor cells (‘seed’) and the environment of the host organ (‘soil’) [25]. Ample preclinical and clinical data has been found in support of the “organ-preference pattern of tumor metastasis”, with the most common sites of metastasis being bone, brain, liver and lung [26]. In a recent population-based analysis, five different metastatic clinical prognostic phenotypes were developed: Stage IV-A was defined as nearly-exclusive bone-only metastases, IV-B as predominant lung metastases, IV-C as predominant liver/lung metastases, IV-D as bone/liver/lung metastases predominant over brain, and IV-E as brain/lung metastases over bone/liver [3]. Using a latent class analysis, the authors were not only able to show that these phenotypes come with statistically different overall survival probabilities, but also that they were at least partly determined by tumor histology. As the authors did not distinguish between oligo- and polymetastatic disease states, such an endeavor seems worthwhile going forward. The MSK-MET cohort represents the largest effort to date to genomically characterize metastatic patterns by prospective clinical sequencing 25,000 patients. The authors also found a strong correlation between genomic alterations such as chromosomal instabilities or copy-number alternation patterns and clinical metastatic patterns and specific target organs in some tumor types [20].

Lastly, the bimodal distribution of either exhibiting a solitary metastasis or >10 metastases is in agreement with the fact that metastasis is an active, highly complex process [27]. Tumor cells have to master the entire metastatic cascade in order to develop from “immature cancer” cells to “overt metastases” [28], [29]. If only a limited number of distant metastases are present, cells have not yet gained comprehensive metastatic capabilities, resulting in a process of metastasis which is fairly inefficient [30]. Yet once many tumor cells have become successful metastatic cells, polymetastasis prevails [31].

The large database of consecutive PET imaging scans as a basis for the assessment of polymetastatic patients constitutes a great strength of this study. One limitation of this study consists in the fact that the review and assessment of imaging reports of polymetastasized patients can be challenging. Even though PET scans were reviewed in cases where imaging reports were inconclusive, counting lesions in polymetastasized patients can be ambiguous, and small and/or non-avid metastases might have been missed, even though PET scans are known to be diagnostically accurate and reliable cross-sectional imaging modalities [32]. Retrospective single-center studies are always biased towards the expertise of the cancer center and, with respect to this study, histologies which are commonly followed with PET imaging. Other shortcomings consist in having lumped different PET tracers and cancers together in a single study as well as having grouped patients with more than ten metastases into one category. While this might have resulted in a bias of the number of patients with a certain number of extracranial distant metastases (PET imaging is not used for detecting brain metastasis), we believe that this did not fundamentally alter the nature of our findings. Such challenges might be solved with the help of automated, machine-learning-based approaches for detecting and counting metastases in the near future [33]. In addition, further comprehensive genomic studies will be required to improve our understanding of the cellular mechanisms underlying the metastatic burden and organ-specific metastases (“organotropism”).

In conclusion, in reviewing the metastatic tumor burden of 1,155 consecutive cancer patients via a PET imaging report review, a statistically unexpected disease pattern was found, which supports both the spectrum theory of metastasis, and the seed and soil hypothesis: The number of distant metastases showed a strong bimodal distribution of the metastatic burden with 26 % of patients having one solitary metastasis, little patients having two to ten metastases, and 43 % of patients harboring >10 metastases. Yet even in patients with >10 distant metastases, the pattern of metastases distribution was restricted to one or two organs in approximately two thirds of patients. These findings further conceptually underpin the currently ongoing clinical trials investigating the addition of locally ablative therapies to standard-of-care in metastatic cancer patients.

## Funding

Sebastian M. Christ received support through the “Young Talents in Clinical Research” Beginner’s Grant from the Swiss Academy of Medical Sciences (SAMW) and the Bangerter-Rhyner Foundation. This project was also supported by the SNF project “CRSII5_183478” as well as the “CCCZ Oligometastatic Disease Program – OMD^ZH^”.

## Authors' contributions

All authors made important contributions to this project. The idea and conceptualization of the project were developed by MG and SMC. SMC obtained the ethical approval for the project. UJM extracted all imaging scans and reports. KP screened all imaging reports. SMC, PH, AH and MG quality-checked the analysis, and KP as well as SMC conducted the data analysis together. KP and SMC prepared the manuscript. Critical input was received from UJM, JW, PH, AH, MA, LA, PB, MM, NA, NZ, and MH. The final version of the manuscript was approved by all authors before journal submission.

## Ethics approval

This retrospective study was approved by the Swiss Cantonal Ethics Committee before the initiation of the project (BASEC ID # 2018-01794).

## Prior publication

The abstract of this study was presented at ESTRO 2023.

## Declaration of competing interest

PB cited research grants to the institution from ViewRay Inc. (Mountain View, CA, USA). NA has received grants from ViewRay Inc. and BrainLab and personal fees from AstraZeneca, Debiopharm, ViewRay and BrainLab, and non-financial support from ViewRay, all outside of the submitted work. MH has received research support from GE Healthcare, a fund by the Alfred and Annemarie von Sick legacy for translational and clinical cardiac and oncological research, and a grant by the Clinical Research Priority Program (CRRP) “Artificial Intelligence in oncological Imaging” of the University Zurich, all outside of the submitted work.
